# The Central Biobank and Virtual Biobank of BIOMARKAPD: A Resource for Studies on Neurodegenerative Diseases

**DOI:** 10.3389/fneur.2015.00216

**Published:** 2015-10-15

**Authors:** Babette L. R. Reijs, Charlotte E. Teunissen, Nikolai Goncharenko, Fay Betsou, Kaj Blennow, Inês Baldeiras, Frederic Brosseron, Enrica Cavedo, Tormod Fladby, Lutz Froelich, Tomasz Gabryelewicz, Hakan Gurvit, Elisabeth Kapaki, Peter Koson, Luka Kulic, Sylvain Lehmann, Piotr Lewczuk, Alberto Lleó, Walter Maetzler, Alexandre de Mendonça, Anne-Marie Miller, José L. Molinuevo, Brit Mollenhauer, Lucilla Parnetti, Uros Rot, Anja Schneider, Anja Hviid Simonsen, Fabrizio Tagliavini, Magda Tsolaki, Marcel M. Verbeek, Frans R. J. Verhey, Marzena Zboch, Bengt Winblad, Philip Scheltens, Henrik Zetterberg, Pieter Jelle Visser

**Affiliations:** ^1^Department of Psychiatry and Neuropsychology, School for Mental Health and Neuroscience, Alzheimer Center Limburg, Maastricht University Medical Center, Maastricht, Netherlands; ^2^Neurochemistry Laboratory and Biobank, Department of Clinical Chemistry, VU University Medical Center, Amsterdam, Netherlands; ^3^Integrated Biobank of Luxembourg, Luxembourg, Luxembourg; ^4^Clinical Neurochemistry Laboratory, Department of Neuroscience and Physiology, Sahlgrenska University Hospital, The Sahlgrenska Academy at University of Gothenburg, Mölndal, Sweden; ^5^Center for Neuroscience and Cell Biology, Institute for Biomedical Imaging and Life Sciences, Faculty of Medicine, University of Coimbra, Coimbra, Portugal; ^6^German Center for Neurodegenerative Diseases (DZNE) e.V. Clinical Neuroscience and Biomarkers, Bonn, Germany; ^7^Laboratory of Alzheimer’s Neuroimaging and Epidemiology, IRCCS Fatebenefratelli, Brescia, Italy; ^8^Department of Neurology, Akershus University Hospital, Lørenskog, Norway; ^9^Institute of Clinical Medicine, University of Oslo, Oslo, Norway; ^10^Department of Geriatric Psychiatry, Central Institute of Mental Health, Medical Faculty Mannheim, University of Heidelberg, Mannheim, Germany; ^11^Department of Neurodegenerative Disorders, Mossakowski Medical Research Centre, Polish Academy of Sciences, Warsaw, Poland; ^12^Behavioural Neurology and Movement Disorders Unit, Department of Neurology, Istanbul Faculty of Medicine, Istanbul University, Istanbul, Turkey; ^13^Neurochemistry Unit, Division of Cognitive and Movement Disorders, 1st Department of Neurology, National and Kapodistrian University of Athens, Athens, Greece; ^14^Department of Neurology, Slovak Medical University, University Hospital Bratislava, Bratislava, Slovakia; ^15^Institute of Neuroimmunology, Slovak Academy of Sciences, Bratislava, Slovakia; ^16^Division of Psychiatry Research, University of Zurich, Schlieren, Switzerland; ^17^Laboratoire de Biochimie Protéomique Clinique, INSERM U1183, Institut de Médecine Régénérative et Biothérapies, CHRU de Montpellier, Université de Montpellier, Montpellier, France; ^18^Department of Psychiatry and Psychotherapy, Universitätsklinikum Erlangen and Friedrich-Alexander Universität Erlangen-Nürnberg, Erlangen, Germany; ^19^Department of Neurodegeneration Diagnostics, Medical University of Bialystok, Bialystok, Poland; ^20^Memory Unit, Department of Neurology, Hospital de la Santa Creu i Sant Pau-Biomedical Research Institute Sant Pau, Barcelona, Spain; ^21^Centro de Investigación Biomédica en Red de Enfermedades Neurodegenerativas (CIBERNED), Madrid, Spain; ^22^Department of Neurodegeneration, Hertie Institute for Clinical Brain Research, University of Tübingen, Tübingen, Germany; ^23^German Center for Neurodegenerative Diseases (DZNE), University of Tübingen, Tübingen, Germany; ^24^Faculty of Medicine, University of Lisbon, Lisbon, Portugal; ^25^Medical Gerontology, School of Medicine, Trinity College Dublin, Dublin, Ireland; ^26^ICN Hospital Clinic i Universitari, Institut d’Investigacions Biomèdiques August Pi i Sunyer, Barcelona, Spain; ^27^Paracelsus-Elena-Klinik, Kassel, Germany; ^28^Department of Neurosurgery and Institute of Neuropathology, University Medical Center Göttingen, Göttingen, Germany; ^29^Section of Neurology, Centre for Memory Disturbances, University of Perugia, Perugia, Italy; ^30^Laboratory for CSF Diagnostics, Department of Neurology, University Medical Centre, Ljubljana, Slovenia; ^31^Department of Psychiatry and Psychotherapy, University Medical Center Göttingen and Translational Dementia Research Group, German Center for Neurodegenerative Diseases (DZNE), Göttingen, Germany; ^32^Danish Dementia Research Centre, Rigshospitalet, Copenhagen University Hospital, Copenhagen, Denmark; ^33^Unit of Neuropathology, Department of Diagnostics and Technology, IRCCS Foundation “Carlo Besta” Neurological Institute, Milan, Italy; ^34^3rd Department of Neurology, Aristotle University of Thessaloniki, Thessaloniki, Greece; ^35^Department of Neurology, Radboud Alzheimer Centre, Donders Institute for Brain, Cognition and Behaviour, Radboud University Medical Centre, Nijmegen, Netherlands; ^36^Department of Laboratory Medicine, Radboud Alzheimer Centre, Donders Institute for Brain, Cognition and Behaviour, Radboud University Medical Centre, Nijmegen, Netherlands; ^37^Research-Scientific-Didactic Centre of Dementia-Related Diseases, Wrocław Medical University, Scinawa, Poland; ^38^Division of Neurogeriatrics, Center for Alzheimer Research, Department of Neurobiology, Care Sciences and Society, Karolinska Institutet, Huddinge, Sweden; ^39^Department of Neurology, Alzheimer Center, VU University Medical Center, Amsterdam, Netherlands; ^40^Department of Psychiatry and Neurochemistry, Institute of Neuroscience and Physiology, The Sahlgrenska Academy at the University of Gothenburg, Mölndal, Sweden; ^41^UCL Institute of Neurology, London, UK

**Keywords:** biobank, cerebrospinal fluid, dementia, Alzheimer’s disease, Parkinson’s disease, neurodegenerative disorders, body fluids

## Abstract

Biobanks are important resources for biomarker discovery and assay development. Biomarkers for Alzheimer’s and Parkinson’s disease (BIOMARKAPD) is a European multicenter study, funded by the EU Joint Programme-Neurodegenerative Disease Research, which aims to improve the clinical use of body fluid markers for the diagnosis and prognosis of Alzheimer’s disease (AD) and Parkinson’s disease (PD). The objective was to standardize the assessment of existing assays and to validate novel fluid biomarkers for AD and PD. To support the validation of novel biomarkers and assays, a central and a virtual biobank for body fluids and associated data from subjects with neurodegenerative diseases have been established. In the central biobank, cerebrospinal fluid (CSF) and blood samples were collected according to the BIOMARKAPD standardized pre-analytical procedures and stored at Integrated BioBank of Luxembourg. The virtual biobank provides an overview of available CSF, plasma, serum, and DNA samples at each site. Currently, at the central biobank of BIOMARKAPD samples are available from over 400 subjects with normal cognition, mild cognitive impairment (MCI), AD, frontotemporal dementia (FTD), vascular dementia, multiple system atrophy, progressive supranuclear palsy, PD, PD with dementia, and dementia with Lewy bodies. The virtual biobank contains information on over 8,600 subjects with varying diagnoses from 21 local biobanks. A website has been launched to enable sample requests from the central biobank and virtual biobank.

## Introduction

There is an urgent need for biomarkers facilitating diagnosis of Alzheimer’s disease (AD) and Parkinson’s disease (PD) at an early stage in the disease course before the onset of clinical symptoms and to predict disease progression. For AD, the 42 amino acid form of β-amyloid (Aβ42) reflecting Aβ deposition in plaques, total tau (T-tau) reflecting the intensity of neuroaxonal degeneration, and phosphorylated tau (P-tau) reflecting the amount of brain tangle pathology are promising cerebrospinal fluid (CSF) biomarkers for early detection (1), but they do not cover all the neurodegenerative processes involved. For PD and dementia with Lewy bodies (DLB), no diagnostic or prognostic CSF or blood biomarkers exist, except for α-synuclein in CSF (2). The use of Aβ42, tau proteins, and α-synuclein for the diagnosis and prognosis of AD and PD is challenged by the high intra- and inter-center variability in biomarker concentration measurements (3–5). The variability in measurements is likely caused by differences in pre-analytical and analytical protocols for sample collection, sample handling, and local assay handling (3, 6–10), as well as by inconsistencies in kit production with batch-to-batch and even within-plate variation (11, 12).

Biomarkers for Alzheimer’s and Parkinson’s Disease (BIOMARKAPD) was a European multicenter study, funded by EU Joint Programme-Neurodegenerative Disease Research (JPND), designed to standardize the assessment of existing assays and to validate novel fluid biomarkers for AD and PD. To support these objectives, BIOMARKAPD has established a central biobank and a virtual biobank for neurodegenerative diseases. Samples for the central biobank have been collected and handled according to standardized operating procedures (13). The virtual biobank provides an overview of the local sample stock at each site. In this article, we will give an overview of clinical data, availability of samples, and the methods for sample collection and processing. Finally, we will explain the procedures for requesting samples.

## Materials and Methods

### Central biobank

#### Study Population

Inclusion criteria for subjects in the central biobank of BIOMARKAPD were a diagnosis of normal cognition, mild cognitive impairment (MCI), AD, PD, dementia with Lewy bodies (DLB), frontotemporal dementia (FTD), vascular dementia (VaD), progressive supranuclear palsy (PSP), multiple system atrophy (MSA), or another type of dementia. Subjects were required to be at least 55 years old (in the MCI group) or at least 40 years old (in all other diagnostic groups). Subjects with normal cognition were clinically evaluated and were required to score above the 10th percentile on the age and education corrected mini-mental state examination (MMSE) (14). MCI was defined as referral to a memory clinic because of cognitive complaints in the absence of dementia. MCI subtypes could be defined *post hoc* based on neuropsychological test performance or CDR score. Subjects with PD were clinically diagnosed according to the UKPDBB criteria (15) or Gelb criteria (16). Subjects with dementia had a minimum score of 18 on the MMSE and were clinically diagnosed according to the NINCDS-ADRDA criteria for probable or possible AD (17), Neary criteria for FTD (18), NINDS-AIREN criteria for VaD (19), and McKeith criteria for DLB (20). Exclusion criteria for all subjects were contra-indications for lumbar puncture and other obvious causes of cognitive impairment such as strokes, severe depression, or endocrine disorders.

### Clinical data

The central biobank collected information on age, gender, education, clinical history [e.g., diagnosis, medication use, a selection of co-morbid disorders (cardiovascular, cerebrovascular, neurological, endocrine, somatic, and psychiatric disorders)], smoking habits and alcohol intake, physical examination [i.e., blood pressure, height, weight, and body mass index (BMI)], general cognition (CDR and MMSE), neuropsychological test performance for the domains of memory, fluency, visuospatial construction, attention, and executive functioning (expressed as raw scores and as *z*-scores according to local norms corrected for age, gender, and education), procedures for sample collection and processing, and the availability of imaging data (e.g., MRI, PET). Clinical data were collected within a timeframe of 6 months around blood/CSF collection.

### Standardized operating procedures

Samples for the central biobank were collected according to defined biobanking pre-analytical standard operating procedures (SOPs) of the BIOMARKAPD project. For CSF collection, processing, and storage, we adhered to the BIOMARKAPD SOP published by del Campo et al. (13). For plasma and serum samples, we adhered to the biobanking guidelines published by Teunissen et al. (21). In addition, we recommended a 60 min minimum clotting time for blood for serum samples in accordance with the instructions of the tube manufacturer. For blood for DNA samples, we recommended storage at maximal −20°C consistent with the guidelines by Teunissen et al. (22). Centers were asked to report deviations from the SOP.

### Sample collection, processing, and storage

Tubes for sample collection and storage were distributed by Integrated BioBank of Luxembourg (IBBL). Blood samples were collected in the following polypropylene tubes: 10 mL EDTA [Becton, Dickinson and Company (BD), ref. 367525] for plasma, 4 mL EDTA (BD, ref. 368861) for whole blood, and 10 mL clot activator tubes (CAT) (BD, ref. 367896) for serum. CSF was collected in 10 mL polypropylene tubes (Sarstedt, ref. 62.610.018). Blood samples for DNA were not centrifuged and stored at maximal −20°C. All other samples were centrifuged at room temperature at 2,000 × *g* (min 1,800 × *g*, max 2,200 × *g*) and stored at −80°C. A maximum of 2 h was allowed between collection and freezing. A more detailed description of the SOP used for the collection of samples for the central biobank can be found elsewhere (13). For every subject 2 mL CSF, 2 mL serum, and 2 mL plasma were stored in 0.5 aliquots (in 0.5 mL Matrix 2D Thermo tubes) and 4 mL blood was stored for DNA isolation. Primary specimens and samples derivatives were coded with a three-letter center code and a subject number. Samples were at first stored locally, and then shipped on dry ice to IBBL for long-term storage. DNA extraction was performed at the IBBL. Samples and associated data were processed and stored at IBBL in compliance with ISO 9001:2008, NF S96-900: 2011, and ISO 17025:2005 standards and the ISBER Best Practices.

### Virtual biobank

The virtual biobank provides an estimation of the number of samples, and clinical (i.e., age, gender, education, CDR scores, MMSE scores, Parkinson scales, neuropsychological test results, information on medication use, and co-morbid disorders) and other biomarker data (i.e., MRI data, amyloid PET, dopamine SPECT) available at each center of subjects with normal cognition, MCI, AD, PD, PD with dementia, DLB, FTD, VaD, PSP, MSA, and other types of dementia. Retrospectively collected samples had been collected according to the center’s own SOPs. Centers that changed to the standardized BIOMARKAPD SOP during the project reported the transition date. All samples remained stored on site.

### Ethics

Centers received approval from their local Ethical Committee and all subjects provided informed consent. All human research was conducted in accordance with the principles of the Declaration of Helsinki.

## Results

### Central biobank

Sample collection for the central biobank was performed in the period October 2013–December 2015. A total of 14 European centers have contributed samples and data to the central biobank. Currently, the central biobank database contains clinical information on 419 subjects, of which 49 had normal cognition, 117 MCI, 164 AD, 24 FTD, 3 VaD, 11 DLB, 25 PD, 5 PD with dementia, 3 PSP, 1 MSA, and 18 other types of dementia (i.e., either unknown or mixed pathology). From almost all subjects CSF samples (*n* = 410), plasma samples (*n* = 413 subjects), serum samples (*n* = 414), and DNA samples (*n* = 414) are available at the central biobank. At the local sites, MRI imaging data are available from 299 subjects, SPECT from 6 subjects, amyloid PET from 14 subjects, and FDG-PET from 28 subjects. Table 1 lists demographic information, neuropsychological tests results, and available imaging data according to diagnostic group. At least 1 neuropsychological test result was available from 307 subjects. The deviations reported from the SOP are shown in Table 2. The most common deviation (82%) was the use of a different needle than the 25G atraumatic needle. For most lumbar punctures, this needle was unavailable (*n* = 239), it was impossible to collect CSF with this needle (*n* = 19) or the neurologist preferred a traumatic needle (*n* = 79). None of the samples had more than the maximum of two freeze and thaw cycles, while 12% of the CSF samples, 1% of the plasma samples, and 13% of the serum samples underwent one freeze and thaw cycle. If the deviation related to needle use and number of freeze and thaw cycles was not taken into account, adherence to the BIOMARKAPD SOP was 91% for CSF collection and centrifugation, 96% for plasma collection and centrifugation, 93% for serum collection and centrifugation, and 100% for DNA collection and processing.

**Table 1:** **Central biobank subject characteristics, *z*-scores on neuropsychological tests, and biomarker data available according to diagnostic group**.

	Total (*n* **=** 419)	Normal cognition (*n* **=** 49)	MCI (*n* **=** 117)	AD (*n* **=** 164)	FTD (*n* **=** 24)	VaD (*n* **=** 3)	DLB (*n* **=** 11)	PD (*n* **=** 25)	PD with dementia (*n* **=** 5)	PSP (*n* **=** 3)	MSA (*n* **=** 1)	Other dementia (*n* **=** 18)
**Demographics**, ***n***	419	49	117	164	24	3	11	25	5	3	1	18
Age, mean (SD)	68.0 (9.3)	62.5 (9.9)	67.1 (9.2)	70.6 (8.5)	63.8 (7.4)	72.3 (5.5)	75.6 (8.9)	68.0 (7.5)	72.2 (5.9)	54.7 (5.9)	80.0 (0)	65.8 (10.1)
Male, % (*n*)	49 (205)	61 (30)	53 (62)	37 (60)	63 (15)	67 (2)	73 (8)	60 (15)	60 (3)	67 (2)	0 (0)	44 (8)
Education, mean years (SD)	9.9 (3.7)	12.2 (2.9)	10.3 (3.4)	9.6 (3.8)	7.9 (3.4)	7.3 (3.1)	8.3 (3.5)	8.9 (3.3)	11.0 (2.8)	14.0 (3.5)	5.0 (0)	8.9 (3.8)
**MMSE**, ***n***	386	49	109	150	23	3	11	17	5	3	1	15
Mean (SD)	23.9 (5.3)	27.6 (2.6)	27.0 (2.2)	21.1 (5.1)	22.9 (5.6)	25.3 (1.5)	21.1 (6.6)	26.3 (5.5)	22.6 (5.9)	22.3 (3.8)	23.0 (0)	19.1 (7.7)
**CDR overall**, ***n***	283	44	82	113	16	2	4	3	1	3	0	15
Mean (SD)	0.8 (0.5)	0.2 (0.3)	0.5 (0.1)	1.1 (0.4)	1.1 (0.6)	1.0 (0)	0.8 (0.3)	1.7 (1.2)	0.5 (0)	1.0 (0)	–	1.2 (0.7)
**NPA (at least 1*z*-score)**, ***n***	307	45	100	108	17	3	7	10	3	3	0	11
Word list immediate recall	−1.8 (1.5)	−0.3 (1.1)	−1.5 (1.3)	−2.8 (1.2)	−2.8 (1.9)	−1.8 (0.4)	−2.3 (1.2)	−0.4 (2.2)	−	−1.8 (2.0)	−	−2.2 (0.5)
Word list delayed recall	−1.7 (1.4)	−0.7 (0.9)	−1.5 (1.4)	−2.5 (1.1)	−1.7 (1.0)	−2.2 (0.6)	−2.1 (1.7)	0.4 (0.4)	−	−1.4 (1.6)	–	−2.4 (0.6)
Story immediate recall	−1.2 (1.7)	0 (0.9)	−1.3 (2.0)	−2.4 (0.8)	−2.7 (0)	–	–	−3.9 (0)	–	–	–	−2.1 (0.4)
Story delayed recall	−0.8 (1.9)	−0.1 (0.9)	−1.7 (2.0)	−0.2 (3.6)	–	–	–	−4.8 (0)	–	–	–	−2.4 (0)
Fluency	−1.0 (1.4)	−0.5 (1.1)	−0.8 (1.5)	−1.5 (1.2)	−1.6 (1.2)	−1.3 (1.4)	0 (1.4)	−0.9 (0.9)	–	1.0 (2.8)	–	−1.1 (1.2)
Copy figures	−0.7 (1.4)	−1.4 (0.9)	−0.4 (1.4)	−0.9 (1.4)	−1.4 (1.6)	0.8 (0.5)	−0.7 (1.5)	0.4 (1.1)	–	−0.9 (2.2)	–	−1.2 (1.2)
TMTA	−1.2 (1.4)	−0.8 (1.4)	−0.9 (1.3)	−1.6 (1.2)	−1.9 (1.6)	−1.5 (0.6)	−0.2 (1.7)	−0.3 (0.8)	–	1.6 (3.7)	–	−2.5 (0.8)
TMTB	−1.5 (1.7)	−1.0 (1.4)	−1.2 (1.7)	−2.1 (1.6)	−2.4 (1.6)	−2.0 (1.6)	−2.1 (1.3)	1.3 (0.1)	–	1.8 (3.5)	–	−2.0 (1.3)
**Fasted, % (*n*)**	35.0 (140)	4.4. (2)	39.8 (45)	30.7 (47)	54.2 (13)	66.7 (2)	36.4 (4)	72.0 (18)	40.0 (2)	0	100 (1)	35.3 (6)
**Erythrocyte count **>**500/**μ**L, % (*n*)**	5.0 (20)	8.9 (4)	3.5 (4)	7.0 (11)	0	0	0	0	0	0	0	5.9 (1)
**MRI**, ***n*^a^**	299	45	90	110	21	2	3	5	3	3	1	16
**SPECT**, ***n*^a^**	6	0	0	1	0	0	2	1	2	0	0	0
**Amyloid PET**, ***n*^a^**	14	2	1	8	1	0	0	0	0	1	0	1
**FDG-PET**, ***n*^a^**	28	1	6	11	4	0	0	0	0	1	0	5

*MMSE, mini-mental state examination; CDR, Clinical dementia Rating; NPA, neuropsychological assessment; TMT, Trail Making Test; MCI, mild cognitive impairment; AD, Alzheimer’s disease; FTD, frontotemporal dementia; VaD, vascular dementia; DLB, dementia with Lewy bodies; PD, Parkinson’s disease; PSP, progressive supranuclear palsy; MSA, multiple system atrophy*.

*Data are mean (SD), count or valid percent*.

*^a^Not in central biobank, but available at local sites*.

**Table 2:** **Deviations from the SOP reported for samples in the central biobank**.

SOP recommendation	Number of deviations	Reason (number of subjects)
**CSF collection**		
Withdrawal of 10 mL CSF (+2 mL for clinical purposes)	14	Slow flow/flow stopped (2); unknown (7); difficulty with positioning (1); patient did not want to continue (2); impossible, no reason specified (2)
25G atraumatic needle	336	Neurologist preferred traumatic needle (79); atraumatic used, but different diameter: 25G not available (238), impossible with 25G (19)
LP location: intervertebral space L3-L5	0	–
Polypropylene tubes	0	–
Erythrocyte count <500/μL	20	Unknown (20)
**CSF processing**		
Centrifuge at 2,000 × *g* (or between 1,800 and 2,200 × *g*) for 10 min at RT	5	2,000 × *g* centrifuge not available (centrifuged at 1,120 × *g*) (5)
Maximum 2 h between collection and freezing (or temporarily store at 4°C)	1	Delay in sample delivery (1)
Freeze at −80°C	0	–
Maximum of 2 freeze and thaw cycles	0^a^	–
**Blood for plasma, processing**		
Centrifuge at 2,000 × *g* (or between 1,800 and 2,200 × *g*) for 10 min at RT	5	2,000 × *g* centrifuge not available (centrifuged at 1,120 × *g*) (5)
Maximum 2 h between collection and freezing (or temporarily store at 4°C	13	Delay in sample delivery (1); unknown (12)
Freeze at −80°C	0	–
Limit freeze and thaw cycles	0^a^	–
**Blood for serum, processing**		
Centrifuge at 2,000 × *g* (or between 1,800 and 2,200 × *g*) for 10 min at RT	5	2,000 × *g* centrifuge not available (centrifuged at 1,120 × *g*) (5)
Maximum 2 h between collection and freezing (or temporarily store at 4°C)	13	Delay in sample delivery (1); unknown (12)
At least 30 min (but preferably >60 min) between collection and centrifugation	10^b^	Mistake <30 min (10)
Freeze at −80°C	0	–
Limit freeze and thaw cycles	0^a^	–
**Whole blood for DNA, processing**		
Freeze below −20°C	0	–

*SOP, standardized operating procedures; LP, lumbar puncture; RT, room temperature. Data are number of subjects in which a deviation of the SOP occurred*.

*^a^One cycle: CSF (50), plasma (5) and serum (55)*.

*^b^Clotting time: between 30 and 50 min (23) and between 50 and 59 min (35)*.

### Virtual biobank

Currently, 21 centers have contributed data to the virtual biobank of BIOMARKAPD. The virtual biobank contains information on CSF samples from 7,550 subjects, EDTA plasma samples from 8,676 subjects, and serum samples from 8,141 subjects. So far, 11 centers have reported that they followed, or changed to, the BIOMARKAPD SOP for sample collection and processing. Table 3 lists the number of subjects per diagnostic group with CSF, EDTA plasma, and serum samples available.

**Table 3:** **Number of subjects in virtual biobank with CSF, EDTA plasma, and serum samples available according to diagnostic group**.

	CSF	EDTA plasma	Serum
Normal cognition, *n*	890	1,831	1,316
MCI, *n*	1,969	1,894	2,066
AD, *n*	2,420	2,440	2,349
FTD, *n*	612	621	647
VaD, *n*	156	187	151
DLB, *n*	277	282	279
PD	439	720	748
PD with dementia, *n*	157	243	219
PSP, *n*	148	146	115
MSA, *n*	68	57	38
Other dementia, *n*	414	255	213
Total	7,550	8,676	8,141

*CSF, cerebrospinal fluid; MCI, mild cognitive impairment; AD, Alzheimer’s disease; FTD, frontotemporal dementia; VaD, vascular dementia; DLB, dementia with Lewy bodies; PD, Parkinson’s disease; PSP, progressive supranuclear palsy; MSA, multiple system atrophy*.

*Data are number of subjects with CSF, EDTA plasma, or serum samples available*.

## Discussion

As part of BIOMARKAPD, a large central and virtual biobank with body fluids were established from over 9,000 subjects with neurodegenerative disorders. The central biobank contains samples from more than 400 subjects of which nearly 40% have AD. Adherence to the BIOMARKAPD SOP was high (>91%) for the collection and processing of CSF, plasma, and serum and blood samples. The virtual biobank contains CSF samples from over 7,500 subjects, plasma samples from over 8,600 subjects, and serum samples from over 8,100 subjects. Samples for the virtual biobank have been collected according to varying local SOPs. However, so far more than half of the centers have reported adopting the BIOMARKAPD SOP in the course of the project.

### Requesting samples from the central or virtual biobank

Researchers in the field of neurodegenerative disorders interested in requesting samples from the central biobank or from the virtual biobank of BIOMARKAPD are invited to consult the following website: http://jpnd.arone.com/. Requests should meet the objectives of BIOMARKAPD project, i.e., to standardize the assessment of existing assays and to validate novel fluid biomarkers for AD and PD. Sample requests will be evaluated by the Analysis Advisory Board (AAB). Approval from the AAB will depend on scientific quality, whether the sample request meets the objectives of BIOMARKAPD, and sample availability. Furthermore, the sample request must meet the following three criteria. First, the researcher must demonstrate that the analysis complies with local medical ethical standards, for example, by showing regulatory approval of a medical ethical committee (MEC), institutional review board (IRB), or equivalent. Second, technical characteristics of assays such as linearity, recovery, specificity, imprecision, sensitivity, and lot-to-lot variability have already been established and of sufficient performance. Third, prior to the request, the diagnostic or prognostic value of the assay should have been already demonstrated in at least 20 controls and 20 diseased subjects. For the central biobank, fees will apply to cover the costs for sample and data collection, processing, and sample storage. Before shipment a material transfer agreement (MTA) needs to be signed.

For the virtual biobank, individual centers can decide on a case-to-case basis whether or not they would like to provide samples and which conditions will apply. When requesting samples from the virtual biobank, contact details will be provided of centers that are interested in meeting the sample request. Centers may use the MTA from the central biobank for the shipment of samples. Detailed information on the methodology of sample preparation and handling, and available clinical information should be requested directly from the center.

## Conclusion

The central and virtual biobanks of BIOMARKAPD provide access to a large repository of CSF and blood samples for researchers in the field of neurodegenerative disorders, enabling progress in the clinical use of biomarkers for the diagnosis and prognosis of neurodegenerative disorders.

## Conflict of Interest Statement

The authors declare that the research was conducted in the absence of any commercial or financial relationships that could be construed as a potential conflict of interest.

## Supplementary Material

The Supplementary Material for this article can be found online at http://journal.frontiersin.org/article/10.3389/fneur.2015.00216
