# Supplementary material for: The Central Biobank and Virtual Biobank of BIOMARKAPD: A Resource for Studies on Neurodegenerative Diseases
Source: Front Neurol. 2015 Oct 15;6:216. doi: 10.3389/fneur.2015.00216 (PMC4606063; doi:10.3389/fneur.2015.00216)
Supplement: Supplementary file 1 [file table_1.pdf]

Supplementary Table: Centers that have contributed samples to the central biobank and virtual biobank

Center	City	Country	Central biobank	Virtual biobank
Rigshospitalet- Copenhagen University Hospital	Copenhagen	Denmark		Yes
Hôpital ST ELOI	Montpellier	France		Yes
Central Institute of Mental Health	Mannheim	Germany	Yes	
University of Tübingen	Tübingen	Germany	Yes	Yes
Universitätsklinikum Erlangen	Erlangen	Germany		Yes
University Clinic Bonn	Bonn	Germany		Yes
Universitätsmedezin Göttingen	Göttingen	Germany		Yes
Aristotle University of Thessaloniki	Thessaloniki	Greece	Yes	Yes
National and Kapodistrian University of Athens	Athens	Greece		Yes
Mercer's Institute for Successful Ageing, St. James's Hospital	Dublin	Ireland	Yes	Yes
Università di Perugia	Perugia	Italy	Yes	
IRCCS Istituto Centro San Giovanni di Dio Fatebenefratelli	Brescia	Italy		Yes
IRCCS Foundation "Carlo Besta" Neurological Institue	Milan	Italy		Yes
VU university medical center (VUMC)	Amsterdam	Netherlands	Yes	Yes
Maastricht University Medical Center (MUMC)	Maastricht	Netherlands	Yes	Yes
Radboud University Nijmegen Medical Center (RUNMC)	Nijmegen	Netherlands		Yes
Akershus Univiversity Hospital	Lørenskog	Norway		Yes
Wroclaw Medical University	Scinawa	Poland	Yes	Yes
Mossakowski Medical Research Centre Polish Academy of Sciences	Warsaw	Poland	Yes	Yes
University of Coimbra Hospital Center	Coimbra	Portugal	Yes	Yes
Instituto de Medicina Molecular	Lisbon	Portugal	Yes	
Institute of Neuroimmunology	Bratislava	Slovakia	Yes	
University Medical Centre Ljubljana	Ljubljana	Slovenia	Yes	
Hospital de la Santa Creu i Sant Pau	Barcelona	Spain		Yes
ICN Hospital Clinic i Universitari and Pasqual Maragall Foundation	Barcelona	Spain		Yes
Istanbul University, Istanbul Faculty of Medicine	Istanbul	Turkey	Yes	
University of Zurich	Zurich	Switzerland		Yes
